# Mechanical properties and curing kinetics of bio-based benzoxazine–epoxy copolymer for dental fiber post

**DOI:** 10.1186/s40643-023-00684-x

**Published:** 2023-09-16

**Authors:** Phattarin Mora, Sarawut Rimdusit, Panagiotis Karagiannidis, Ukrit Srisorrachatr, Chanchira Jubsilp

**Affiliations:** 1 Department of Chemical Engineering, Faculty of Engineering, Srinakharinwirot University, Nakhonnayok, 26120 Thailand; 2 Center of Excellence in Polymeric Materials for Medical Practice Devices, Department of Chemical Engineering, Faculty of Engineering, Chulalongkorn University, Bangkok, 10330 Thailand; 3 School of Engineering, Faculty of Technology, University of Sunderland, Sunderland, SR6 0DD UK; 4 Department of Medical Services, Institute of Dentistry, Nonthaburi, 11000 Thailand

**Keywords:** Bioresource, Dental glass fiber post, Finite-element analysis, Thermoset polymer

## Abstract

Biocopolymers based on vanillin/fufurylamine–biobenzoxazine (V-fa) and epoxide castor oil (ECO), a bioepoxy, were prepared for application as dental fiber-reinforced composite post. The mechanical and thermal properties of the V-fa/ECO biocopolymers were assessed with regard to the influence of ECO content. The addition of the ECO at an amount of 20% by weight into the poly(V-fa) preserved the stiffness, glass transition temperature and thermal stability nearly to the poly(V-fa). Differential scanning calorimetry (DSC) was used to examine the curing kinetics of the V-fa/ECO monomer system with different heating rates. To determine the activation energy (*E*_*a*_), the experimental data were subjected to the isoconversional methods, namely Flynn–Wall–Ozawa (FWO) and Friedman (FR). The V-fa/ECO monomer mixture showed average *E*_*a*_ values of 105 kJ/mol and 94 kJ/mol. The results derived using the curing reaction model and the experimental data were in good agreement, demonstrating the efficacy of the FWO method for determining the curing kinetics parameters. The simulated mechanical response to external applied loads by finite-element analysis of the tooth model restored with glass fiber-reinforced V-fa/ECO biocopolymer post showed a similar stress field to the tooth model restored with a commercial glass fiber post. Therefore, based on the findings in this work, it is evident that the bio-based benzoxazine/epoxy copolymer possesses a great potential to be used for dental fiber post.

**Graphical Abstract:**

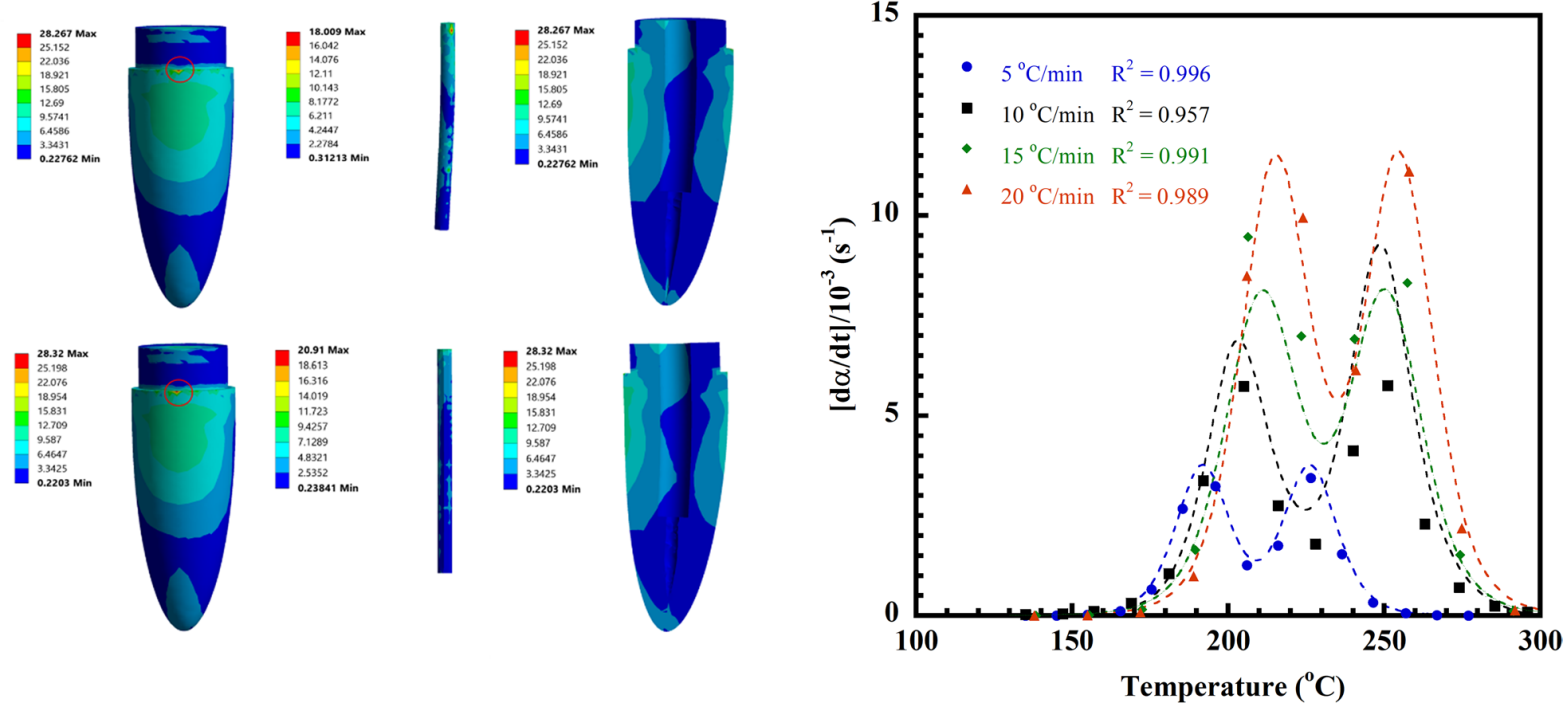

## Introduction

Fiber-reinforced polymer (FRP) composites, i.e., polymer matrix reinforced with fibers, such as glass, carbon, aramid, or natural fibers of plant origin, are commonly used in the construction, marine, automotive, and aerospace industries (Bhardwaj et al. 2021; Birniwa et al. 2023; Shadhin et al. 2021; Shuvo 2020; Zhang et al. 2022; Zulkifli et al. 2016). In recent years, the evolution of FRP composite posts or fiber posts as an alternative to metal and ceramic posts to reinforce tooth treated with endodontics has been investigated (Costa et al. 2022; Genovese et al. 2005; Mora et al. 2022; Ortiz-Magdaleno et al. 2023) due to the increasing demand for tooth-colored posts, the low biocompatibility, the risk of corrosion and the negative aesthetic impact of cast metal posts. Among polymer fiber posts, conventional epoxies acted as the most widely used polymer bases (Fouad et al. 2020). Various amines, anhydrides and acids are commonly used to cure epoxy resins based on diglycidyl ether components. Amines have some drawbacks, such as high toxicity and short pot life (usable time), while anhydrides can be affected by the amount of moisture in the formulation (Cao et al. 2022; Gotro 2022; Hara 1990). Cross-linked epoxy also suffers from low thermal stability and moderate thermal expansion (Kurihara et al. 2012). Consequently, recent studies found other polymers, such as polybenzoxazine (a novel type of phenolic resin) and polyimide to substitute the conventional polymers for fiber posts (Elsubeihi et al. 2020; Mora et al. 2022). Copolymers of an epoxy with a benzoxazine have been reported, where polybenzoxazine forming first, acts as a curing agent for the epoxy; the addition of epoxy to the polybenzoxazine network results in significantly higher glass transition temperature (T_g_) and flexural strength than polybenzoxazine, with only a minimal stiffness loss (Okhawilai et al. 2017; Rao et al. 2005; Rimdusit & Ishida 2000). The curing kinetics of benzoxazine/epoxy system has also been studied by differential scanning calorimetry (Jubsilp et al. 2010; Shutov et al. 2022). It was found that there were two dominant curing processes which were similar to curing processes observed for the conventional epoxy without catalyst (Liu et al. 2009). The activation energy of benzoxazine/epoxy, i.e., 81 kJ/mol and 118 kJ/mol were found to be similar for the epoxy curing under non-isothermal conditions, i.e., 69.7 and 88.7 kJ mol^−1^ based on the Kissinger method, 68.2 and 86.9 kJ/mol based on the Flynn–Wall–Ozawa (FWO) method (Wu et al. 2018). In addition, the no-difference in the curing kinetic model that was autocatalytic kinetic due to the autocatalytic effect of hydroxyl groups generated in the curing reaction of the benzoxazine/epoxy system and the epoxy, has been observed (Jubsilp et al. 2010; Liu et al. 2009).

However, in recent years, with dwindling oil supplies and serious environmental concerns (Leong et al. 2016; Trapé et al. 2021), bioepoxies and biobenzoxazines having similar properties to those of the petroleum bases have been successfully developed from vegetable oils, lignocellulosic biomass (vanillin, eugenol, lignin), furans and tannins. Bioepoxies based on epoxidized vegetable oil such as epoxidized castor oil (ECO), epoxidized soybean oil (ESO), epoxidized linseed oil (ELO) have been synthesized (Park et al. 2004). Castor oil (CO) is an inexpensive vegetable oil extracted from the seeds of the CO plant castor bean (Ricinus communis). The CO's long shelf life, relatively low toxicity, accessible availability, and unique functionality make it superior to other vegetable oils (Sudha et al. 2017). For a novel renewable-based benzoxazine, i.e., V-fa, it has been synthesized without solvents from vanillin produced from lignin and furfurylamine which is also an eco-friendly product derived from various agricultural by-products, such as corncobs, sugar cane bagasse, wheat bran and oats. The formyl group in the bio-based vanillin/furfurylamine benzoxazine helps benzoxazine to cure at low temperatures. The resulting poly(V-fa)-containing furan groups has a high T_g_, excellent thermal properties, and good adhesive properties (Sini et al. 2014). Hence, the efforts for investigation of possibility of the biobenzoxazine/bioepoxy system as a new material base was considered and appreciated. Hombunma et al. (2019) developed V-fa/ECO biocopolymers for shape memory materials under thermal stimulation. The V-fa/ECO biocopolymer with 40 wt% ECO showed good balance between thermo-mechanical properties and shape memory performances. It was found that under NIR light stimulation, the percent weight of ECO in the V-fa/ECO biocopolymer should be at least 50 to achieve a high recovery ratio of 100% within 30 s of NIR irradiation (Amornkitbamrung et al. 2020). In addition, the V-fa/ECO with 50 wt% ECO reinforced with nanofiller such as graphene and multiwalled carbon nanotubes (MWCNT) possess good shape fixity and good shape recovery under NIR actuation (Prasomsin et al. 2019; Srisaard et al. 2021). It is possible that the V-fa/ECO system could be tailor-made to meet the requirements for dental fiber posts.

Therefore, the aim of this work is to achieve a biocopolymer based on bio-based benzoxazine (V-fa) and bio-based epoxy (epoxidized castor oil; ECO) suitable for polymer base dental fiber posts. The effects of ECO contents on curing behavior, mechanical properties and thermal stability were investigated. The thermal curing kinetics of the biocopolymer were also studied by DSC. Its kinetic parameters were evaluated and predicted by the isoconversional methods, i.e., FWO and Friedman. In addition, the mechanical response to external applied loads of tooth models restored with a new glass fiber-reinforced V-fa/ECO biocopolymer post and with a commercial glass fiber post were simulated by finite-element analysis, aiming to provide helpful guidance for dental fiber post application.

## Materials and methods

### Materials

Bio-based vanillin/furfurylamine-based benzoxazine monomer (V-fa) was prepared from vanillin and furfurylamine purchased from Tokyo Chemical Industry Co., Ltd. (Tokyo, Japan) and paraformaldehyde purchased from Merck Co., Ltd. (Darmstadt, Germany). ECO was provided by Aditya Birla Chemicals Thailand Ltd. (Rayong, Thailand). All chemicals were used as received. E-glass fiber plain fabrics with an areal density of 600 g/m^2^ were purchased from Thai Poly Add Ltd. Partnership, Bangkok, Thailand.

### V-fa monomer preparation

The V-fa monomer was prepared from vanillin, paraformaldehyde, and furfurylamine at mol ratios of 1:2:1 using a solventless method (Ishida, 1996). The three reactants were mixed in an aluminum pan at a temperature of 110 °C for 1 h and a low viscosity yellow liquid monomer was obtained.

### V-fa/ECO biocopolymer preparation

**Scheme 1.**
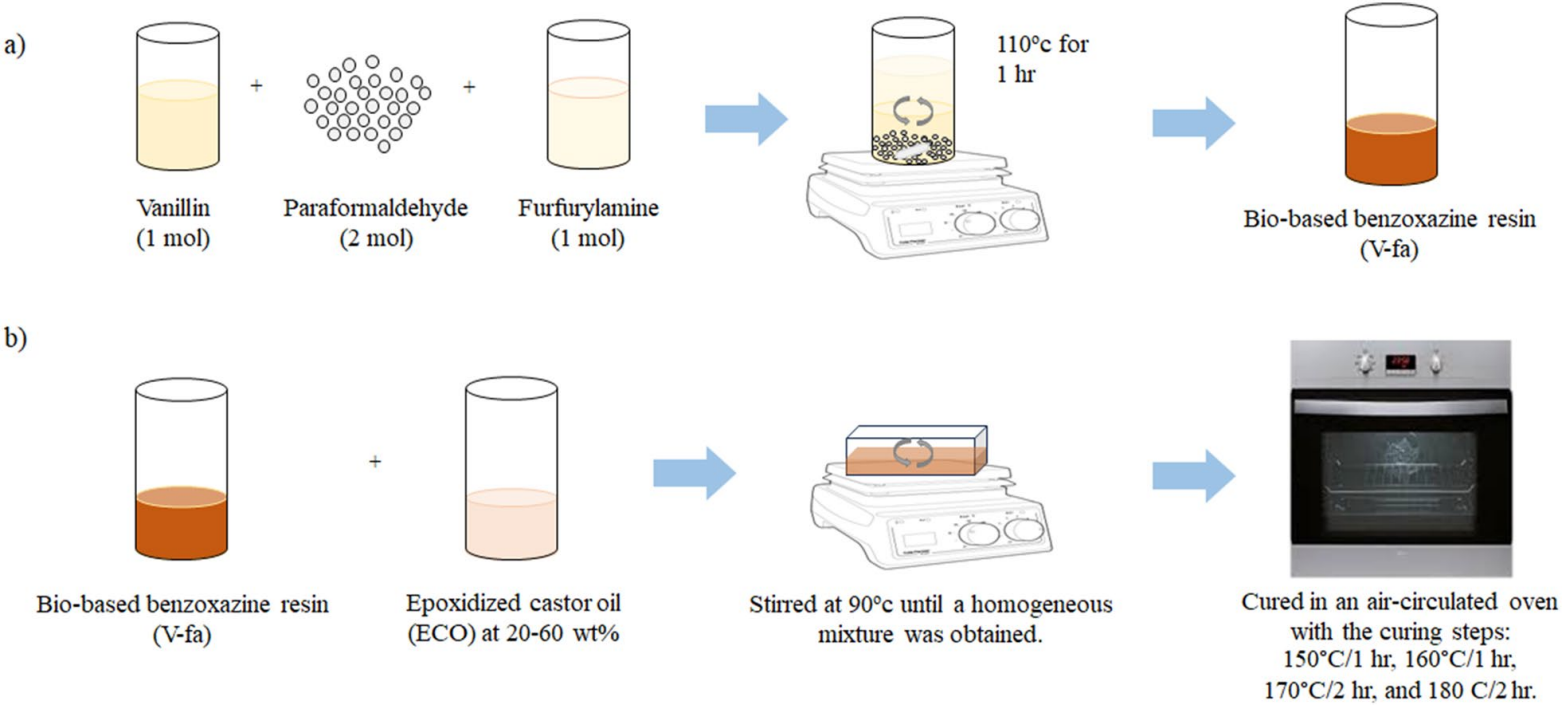
Preparation of **a** V-fa monomer and **b** V-fa/ECO biocopolymer

The V-fa/ECO monomer mixtures were prepared by mixing the V-fa/ECO at a mass ratio of 80/20, 70/30, 60/40, 50/50, and 40/60. Each mixture was heated at 90 °C in an aluminum pan and stirred until a homogeneous mixture was obtained. The mixtures were thermally cured in an air-circulated oven with the curing steps: 150 °C/1 h, 160 °C/1 h, 170 °C/2 h, and 180 °C/2 h. The cured V-fa/ECO biocopolymers were characterized after cooling down to room temperature (Scheme 1).

### Characterization

The curing behavior of the samples was investigated using a differential scanning calorimeter DSC1 Module from Mettler Toledo (Thailand) Ltd. (Bangkok, Thailand). A sample mass of 5–10 mg was sealed in a lidded aluminium pan and heated from 25 °C to 300 °C at heating rates of 5, 10, 15, and 20 °C/min under a nitrogen purge with a flow rate of 50 mL/min.

The chemical structure of the samples was evaluated by Fourier Transform Infrared Spectroscopy (FT-IR) on a Perkin Elmer GX FT-IR spectrometer equipped with an ATR accessory (Perkin Elmer Co., Ltd., Waltham, MA). All spectra were taken with 64 scans at a resolution of 4 cm^−1^ over a spectral range of 4000–650 cm^−1^.

A dynamic mechanical analyzer (model DMA1, Mettler Toledo, Switzerland) was utilized to study dynamic mechanical properties of the samples under three-point bending mode. Distortion was measured at a frequency of 1 Hz with an amplitude of 30 µm. Each sample was heated from 30 °C to 300 °C at a rate of 2 °C/min. The dimensions of the samples were 10 mm × 50 mm × 3 mm.

The degradation temperature of the samples was recorded using a thermogravimetric analyzer (model TGA1 Module Mettler-Toledo, Thailand). Approximately 10 mg of sample was heated from 25 °C to 850 °C at a heating rate of 10 °C/min under nitrogen atmosphere with a flow rate of 50 mL/min.

The flexural properties of the sample were assessed using a universal testing machine, model 8872, Instron (Thailand) Co., Ltd., Bangkok, Thailand. The sample were examined in accordance with ASTM D790M-93 using a 48 mm support span and a 1.2 mm/min crosshead speed. The sample dimension was 25 mm × 60 mm × 3 mm. The five composite samples were tested and the average value were reported.

**Fig. 1:**
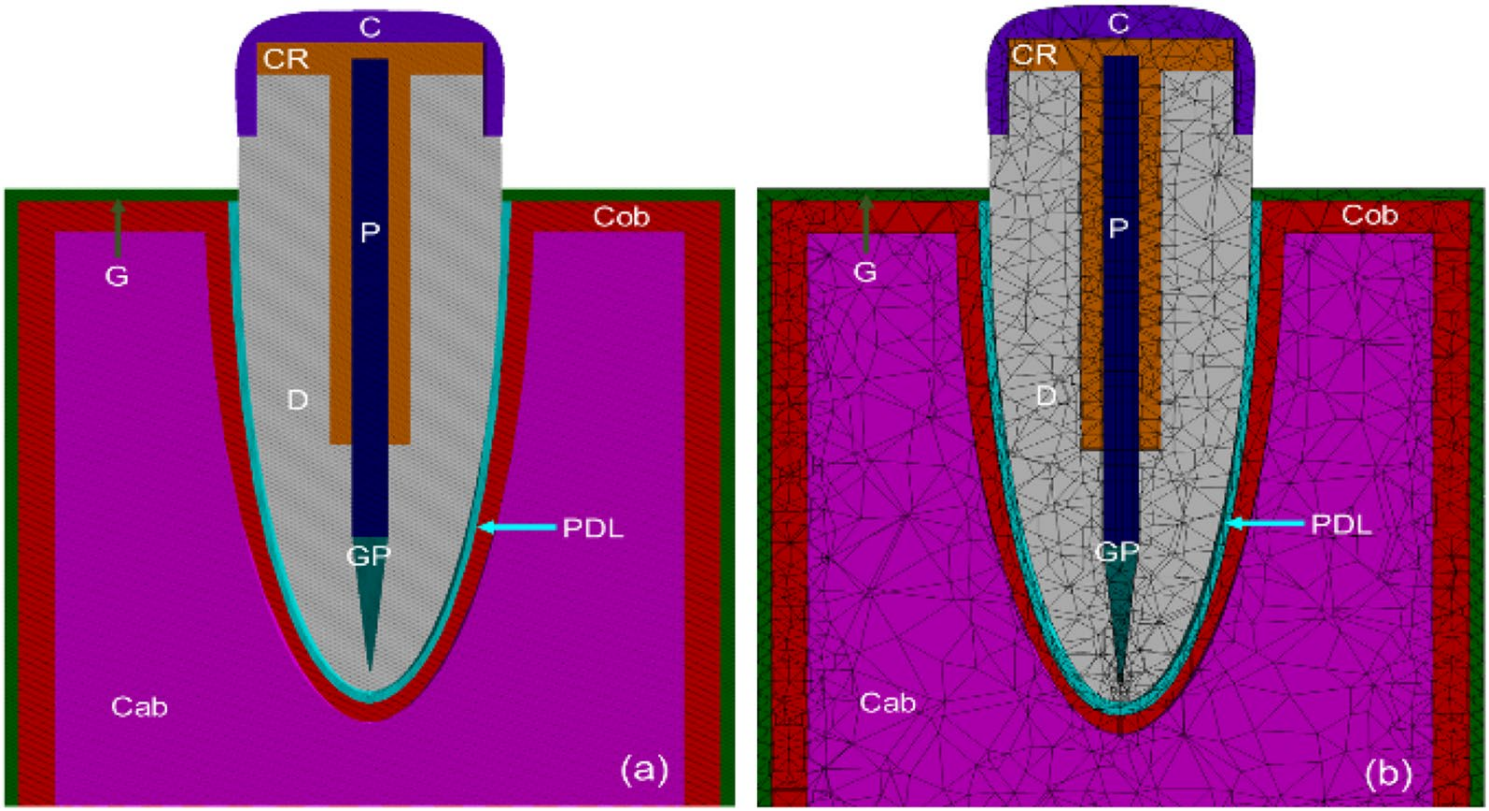
Schematic of tooth model restored with glass fiber post (GF-reinforced V-fa/ECO biocopolymer post); C—porcelain crown, CR—composite resin, P—post, GP—gutta-percha, D—dentin, Cob—cortical bone, PDL—periodontal ligament, Cab—cancellous bone

A 3D model was created in the ANSYS Workbench 2022 R1 (Ansys, Inc. United States) Design Modeller. In Fig. 1, a model of a root canal treated tooth containing all structures (crown, composite resin, glass fiber (GF) reinforced V-fa/ECO biocopolymer post, dentin, gutta-percha) is shown. A 3D mesh was then created using the structurally solid elements defined by the nodes. The numbers of generated elements and nodes for the tooth reconstructed with the post were 112,376 and 208,652, respectively.

## Results and discussion

### Curing behavior of V-fa/ECO monomer mixtures

**Fig. 2:**
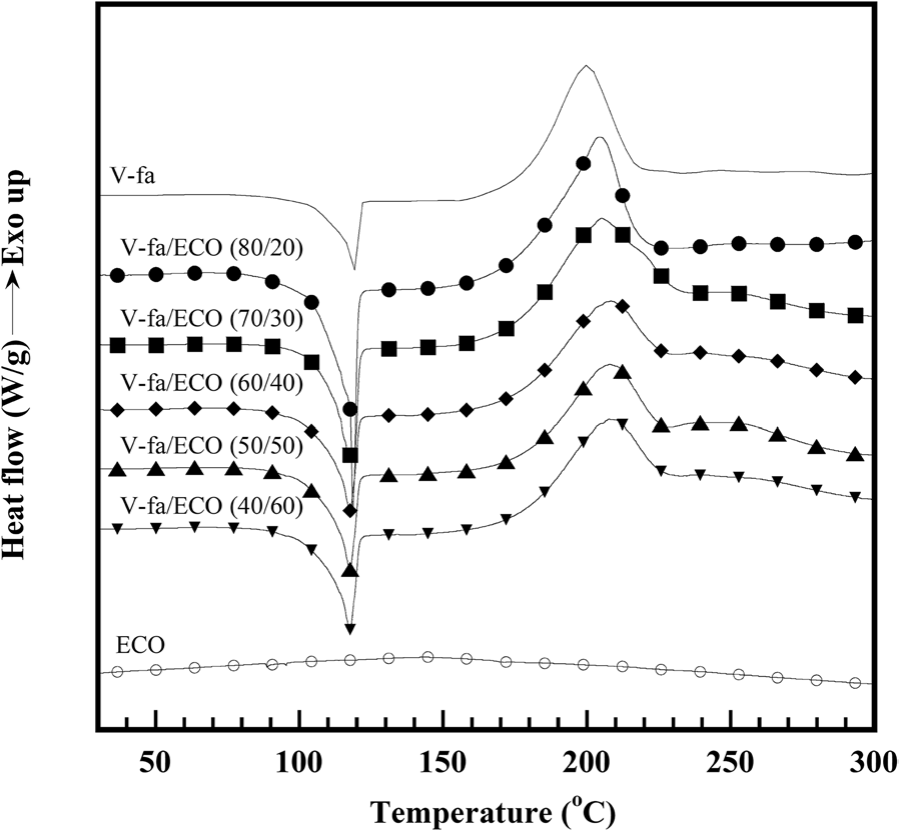
DSC thermograms of V-fa/ECO monomer mixtures at different V-fa/ECO mass ratios

**Fig. 3:**
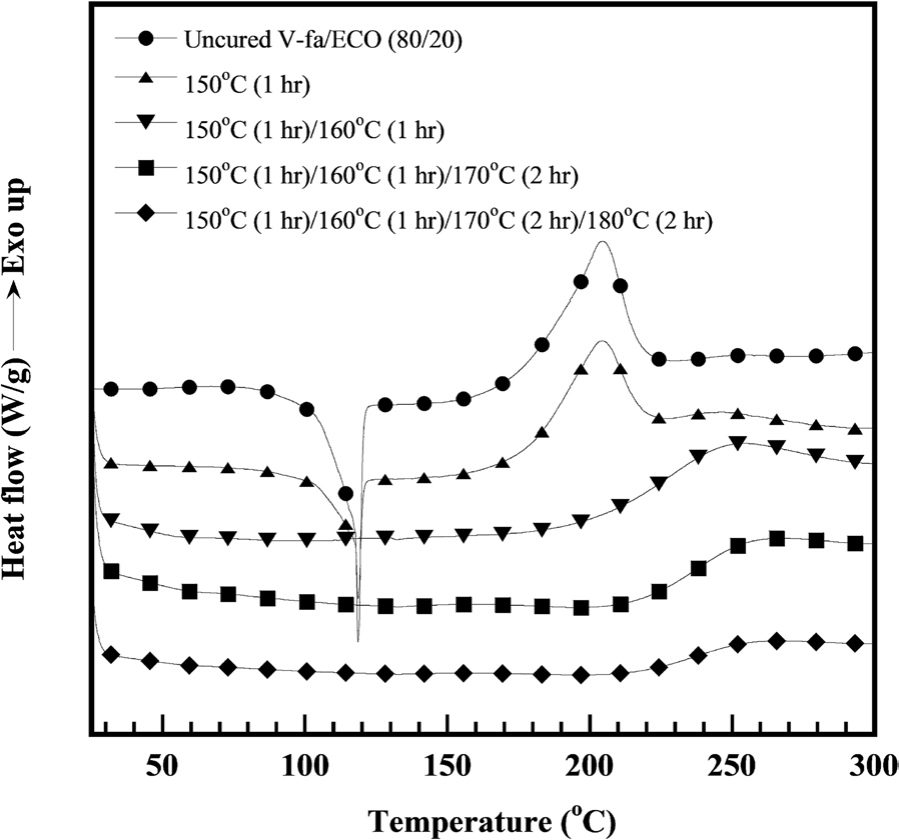
DSC thermogram of V-fa/ECO (80/20) monomer mixture at various curing conditions

Figure 2 shows DSC thermograms recorded at a heating rate of 10 °C/min of V-fa, ECO, and V-fa/ECO monomer mixtures at different V-fa/ECO mass ratios. The curing exothermic peak of the V-fa monomer is approximately at 200 °C, while that of the ECO did not appear. This behavior confirmed that the V-fa monomer can be cured by heat, whereas the ECO requires curing agent as was also observed in the petroleum-based ones (Jubsilp et al. 2010; Shutov et al. 2022). Two low and high temperature curing exothermic peaks were obtained for the V-fa/ECO monomer mixtures. The low-temperature curing exothermic peak (peak-1) of the V-fa/ECO monomer mixture shifted slightly to higher temperatures with increasing ECO content, i.e., 204 °C, 205 °C, 207 °C, 208 °C, and 208 °C for 20, 30, 40, 50, and 60 wt% ECO, respectively, while the increase of the ECO showed no effect on the exothermic curing peak at the higher temperature (peak-2) of all V-fa/ECO monomer mixtures as the exothermic peak temperatures at peak-2 were at about 249 °C. The existence of two exothermic curing peaks illustrated that different curing reaction pathways may occur simultaneously or sequentially. To distinctly confirm two different curing reaction pathways of the V-fa/ECO monomer mixtures, DSC thermograms of uncured V-fa/ECO (80/20) mixture and cured V-fa/ECO (80/20) at various curing conditions were recorded and are presented in Fig. 3. The thermogram of partially cured V-fa/ECO (80/20) at 150 °C for 1 h showed the two exothermic curing peaks and insignificant change in the exothermal peak temperatures, i.e., 200 °C at peak-1 and 249 °C at peak-2. When the partially cured V-fa/ECO (80/20) were continually processed at 160 °C for 1 h, peak-1 was almost disappeared. While, peak-2 still appeared implying that the effect of the curing peak at the lower temperature to the curing peak at the higher temperature could be neglected. Therefore, most probably the curing peak at the lower temperature is due to the oxazine ring opening of the benzoxazine. The higher temperature reaction may be the reaction of the secondary hydroxyl, which was formed during the benzoxazine ring opening reaction reacting with another epoxy ring (Ambrozic et al. 2020).

### Chemical structure of V-fa/ECO biocopolymers

**Fig. 4:**
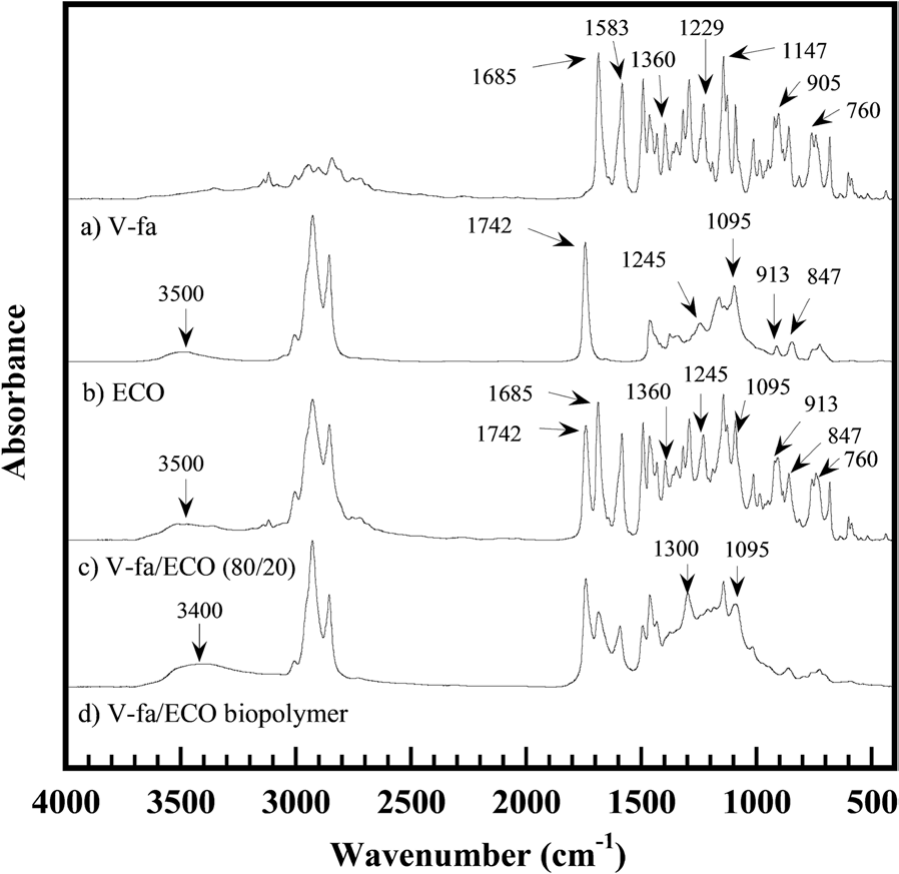
FT-IR spectra of **a** V-fa, **b** ECO, **c** V-fa/ECO monomer mixture, **d** V-fa/ECO biocopolymer

**Fig. 5:**
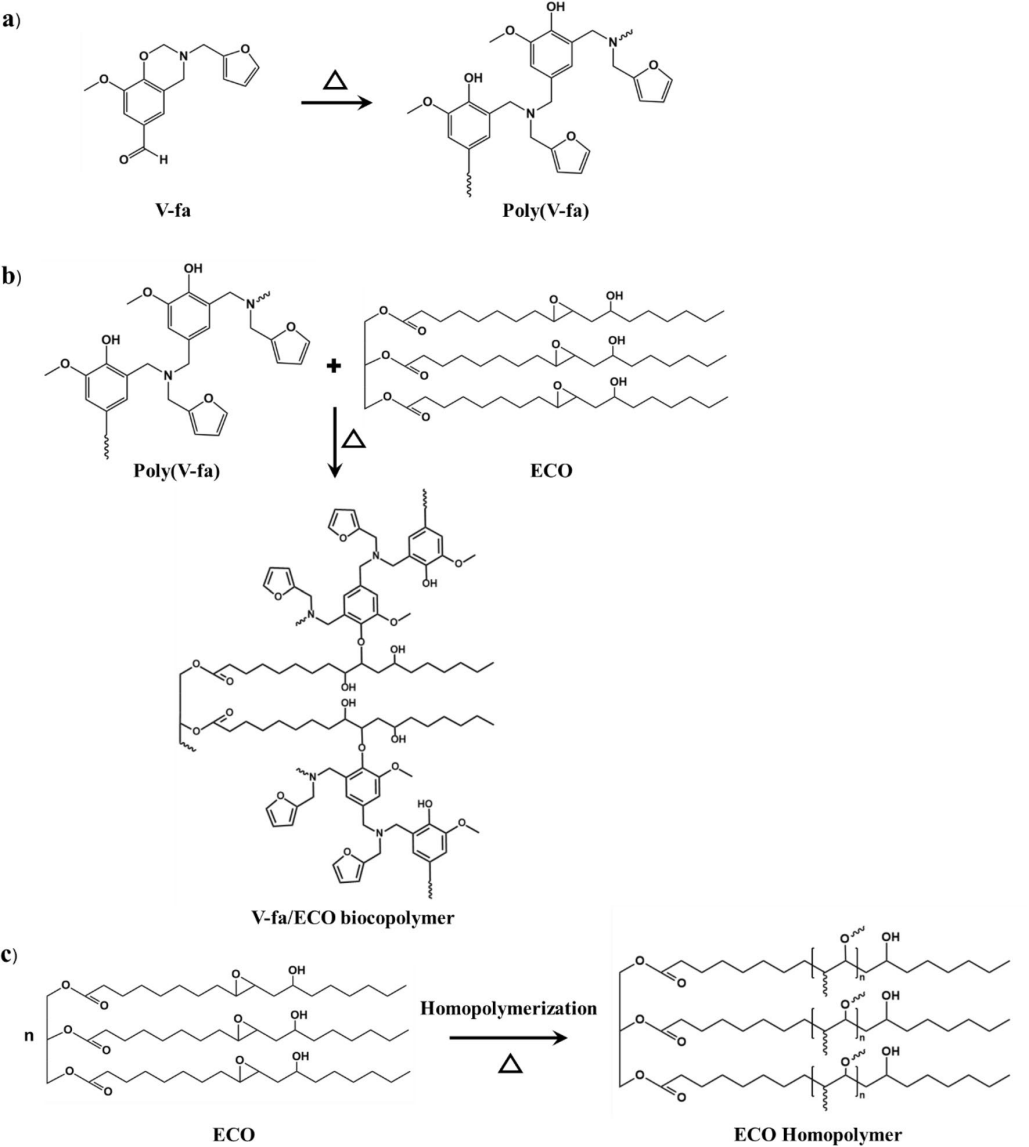
**a** Proposed curing reaction of the V-fa monomer and **b** a plausible chemical reaction between the poly(V-fa) and the ECO **c** homopolymerization of ECO

To understand the chemical reactions during the curing process, FTIR spectra of the V-fa, ECO, V-fa/ECO (80/20) monomer mixture, and V-fa/ECO (80/20) biocopolymer were recorded and are presented in Fig. 4. The bands of the V-fa were observed as follows: 905 and 1229 cm^−1^, and 1147 cm^−1^ are attributed to C–O–C, and C–N–C of oxazine ring, 760 and 1583 cm^−1^ are attributed to furan group, 1360 cm^−1^ is attributed to tetra-substituted benzene ring and 1685 cm^−1^ attributed to carbonyl group (-CHO) (Hombunma et al. 2019; Sini et al. 2014). The bands of the ECO were 847, 913, and 1245 cm^−1^ attributed to oxirane ring, 1095 and 1742 cm^−1^ attributed to C = O stretching and C–O–C stretching of ethers (Sini et al. 2014). The broad band at 3500 cm^−1^ was also observed that was assigned to O–H stretching of hydroxyl groups (de Luca 2009; Dutta et al. 2022, 2020). For the V-fa/ECO monomer mixture, all bands of the V-fa and the ECO appeared. After the curing process, the V-fa/ECO biocopolymer was obtained. A broad peak appeared at about 3200–3800 cm^−1^ and a band at 3400 cm^−1^ attributed to the hydroxyl group formed by the thermal ring-opening reaction of the oxazine ring. Meanwhile, the peak intensity of the oxazine ring disappeared, but new peaks appeared at 1090 and 1300 cm^−1^ attributed to the ether linkages formed by the reaction between the hydroxyl groups of poly(V-fa) and the epoxy groups of ECO, and by the epoxy homopolymerization. Based on the combination of DSC and FTIR analysis results, the chemical reactions for the curing process can be proposed for the V-fa/ECO biocopolymer, as can be seen in Fig. 5. The hydroxyl groups formed due to the oxazine ring opening are presented in Fig. 5a, while the ether linkage formation can be seen in Fig. 5b. In addition, it is possible that ECO homopolymer was also formed at high temperature, as shown in Fig. 5c.

### Dynamic mechanical analysis of V-fa/ECO biocopolymers

**Fig. 6:**
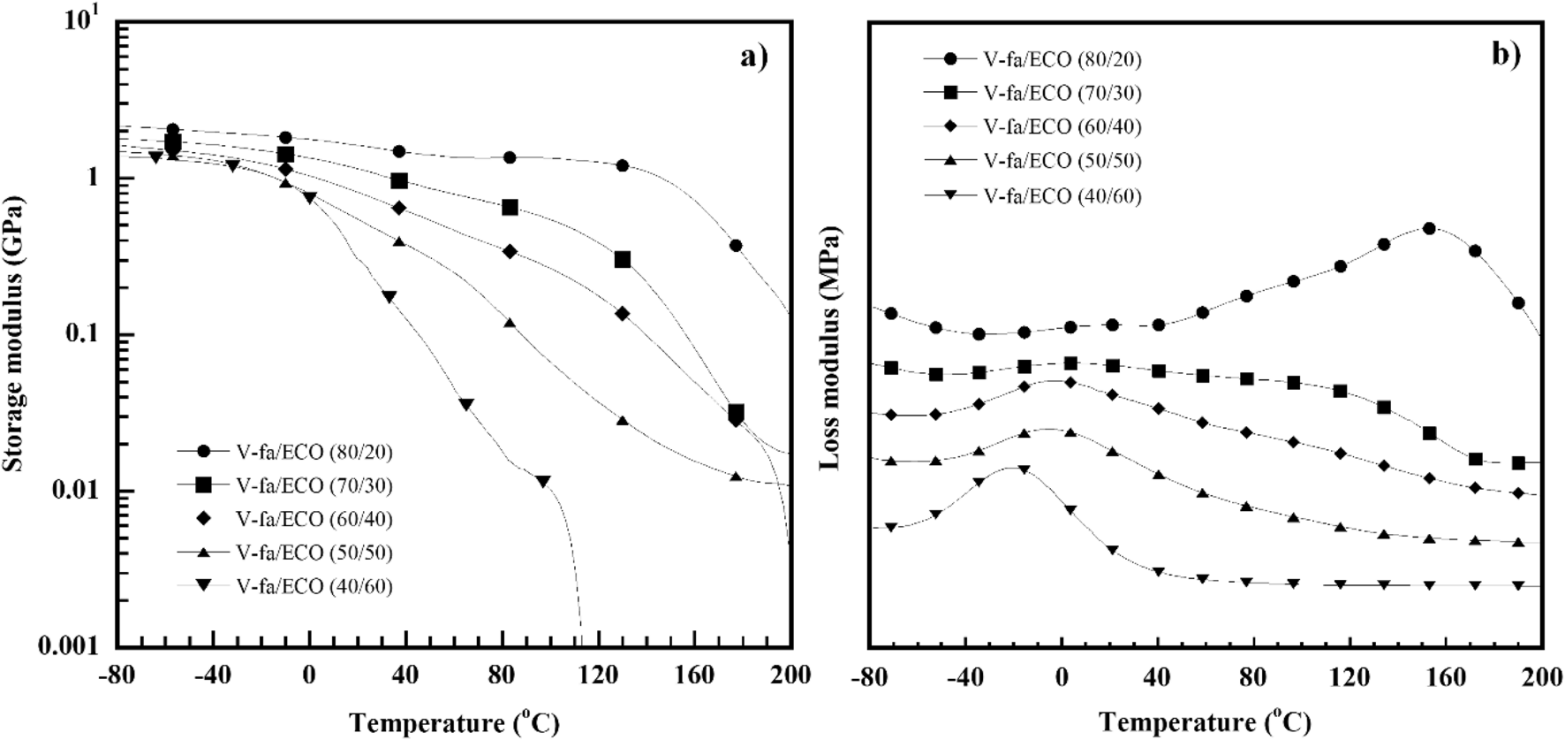
Thermomechanical property of V-fa/ECO biocopolymers at different V-fa/ECO mass ratios **a** storage modulus, **b** loss modulus

Dynamic mechanical properties, i.e., storage or elastic modulus (E′) and loss or viscous modulus (E″) of the V-fa/ECO biocopolymers are plotted in Fig. 6. As shown in Fig. 6a, the E′ of the V-fa/ECO biocopolymers as a function of temperature was improved by increasing portion of the V-fa, as similarly reported by Hombunma et al. (2020). The poly(V-fa) contains benzene rings, providing lower molecular movement than the ECO which contains long alkyl chain providing higher molecular mobility. These results indicated that the V-fa/ECO biocopolymers were innovative materials, where stiffness and flexibility can be tailored by varying the V-fa and the ECO contents. In addition, the higher V-fa content in the V-fa/ECO biocopolymers can improve the dimensional stability, as a sharp drop in E′ in the transition region at higher temperatures was obtained. Therefore, as expected, the T_g_ of the V-fa/ECO biocopolymers was also enhanced with an increase of the V-fa content. In addition, it was observed that the obtained mechanical properties were like those of a conventional petroleum-derived epoxy resin (Saba et al. 2019; Zeng et al. 2015), petroleum-based polybenzoxazine (Jubsilp et al. 2013) and petroleum-based polybenzoxazine/epoxy (Forchetti Casarino et al. 2023).

In Fig. 6b, two distinct T_g_s, i.e., T_g1_ at the lower temperature and T_g2_ at the higher temperature were detected for the V-fa/ECO biocopolymers at 20–50 wt% ECO (T_g1_ = 20 °C, 2 °C, -2 °C, -4 °C and T_g2_ = 158 °C, 130 °C, 107 °C, 98 °C, respectively), while single T_g_ for the V-fa/ECO biocopolymer at 60 wt% ECO was observed, i.e., T_g_ = -18 °C. This characteristic indicated that the ECO domain in the V-fa/ECO biocopolymer significantly affected the T_g1_ value of the biocopolymer, while T_g2_ was the influence from the cross-linked V-fa/ECO domain (Hombunma et al. 2019). In addition, the increase of the T_g1_ and T_g2_ of the V-fa/ECO (80/20) biocopolymer can be expected by reinforcement of glass fiber. It is possible that T_g1_ of the glass fiber-reinforced V-fa/ECO biocopolymer may be increased to reach the service temperature of the human tooth from food consumption, tooth restoration, and dental treatment due to the expected good interaction of the polybenzoxazine matrix to the glass fiber as reported in the previous work (Mora et al. 2022).

### .

### Thermal stability of V-fa/ECO biocopolymers

**Fig. 7:**
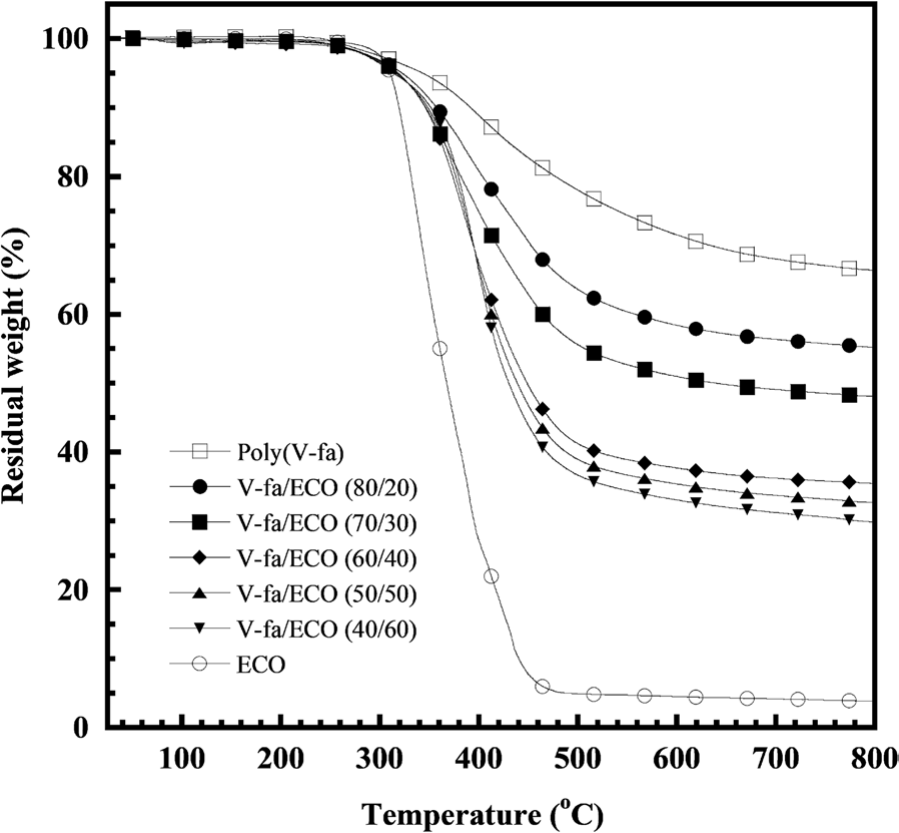
TGA thermogram of V-fa/ECO biocopolymers at various V-fa/ECO mass ratios

Thermal degradation is a type of polymer degradation in which detrimental chemical changes occur at elevated temperatures, changing polymer properties. Therefore, understanding the thermal degradation processes is important because of the proper use of polymers, their storage, and finally recycling. Thermal stability of the V-fa/ECO biocopolymers at various ECO contents was characterized by the degradation temperature at 5% weight loss (T_d5_) as can be seen in Fig. 7. It was found that the V-fa/ECO biocopolymers with 20, 30, 40, 50, and 60 wt% ECO showed the T_d5_ of 323 °C, 318 °C, 317 °C, 314 °C, and 313 °C, respectively, which tended to decrease with an increase ECO content due to lower T_d5_, i.e., 310 °C of the ECO compared to the T_d5_ of the poly(V-fa), i.e., 343 °C. This indicated that the chemical structure of the ECO can be more easily degraded compared to the chemical structure of poly(V-fa) containing phenolic hydroxyls and furan groups. However, the obtained result shows that the degradation temperature of the V-fa/ECO biocopolymers was significantly higher than the service temperature (~ 47 °C) of the human tooth for hot food and drink (Wang et al. 2022). In addition, the T_d5_ of the poly(V-fa)/ECO biocopolymer was similar to other polymers used for fiber posts, such as epoxy (T_d5_ ~ 322.4–355.7 °C) (Nie et al. 2020) and bismaleimide (BMI) (T_d5_ ~ 400 °C) (Chen et al. 2015). In addition, the char yield (CR) at 800 °C of V-fa/ECO biopolymer resulted mainly from the ECO constituent as a decrease in the CR with an increase the ECO content of the V-fa/ECO biocopolymer was observed, i.e., from CR = 55% for V-fa/ECO (80/20) to CR = 30% for V-fa/ECO (40/60). This is because of higher CR of the poly(V-fa), i.e., 66% than that of the ECO, i.e., 3.8%. However, the CR of V-fa/ECO biocopolymer was rather high than epoxy and BMI polymers with the CR of 13–23.5% at 700–800 °C (Li et al. 2015; Nie et al. 2020).

### Non-isothermal curing kinetics of V-fa/ECO system

Curing kinetics is an important aspect of polymer systems as it determines the time available for molding and storage. Among the most chemical reaction systems, the reaction rate is temperature dependent mostly. Studies of curing kinetics are mainly carried out using non-isothermal and isothermal methods. Nonetheless, non-isothermal methods are widely accepted due to the advantage of not requiring prior knowledge of the reaction mechanism, the V-fa/ECO (80/20) monomer mixture was chosen to study curing kinetics, because it showed balanced mechanical and thermal properties for use as a matrix for glass fiber post.

**Fig. 8:**
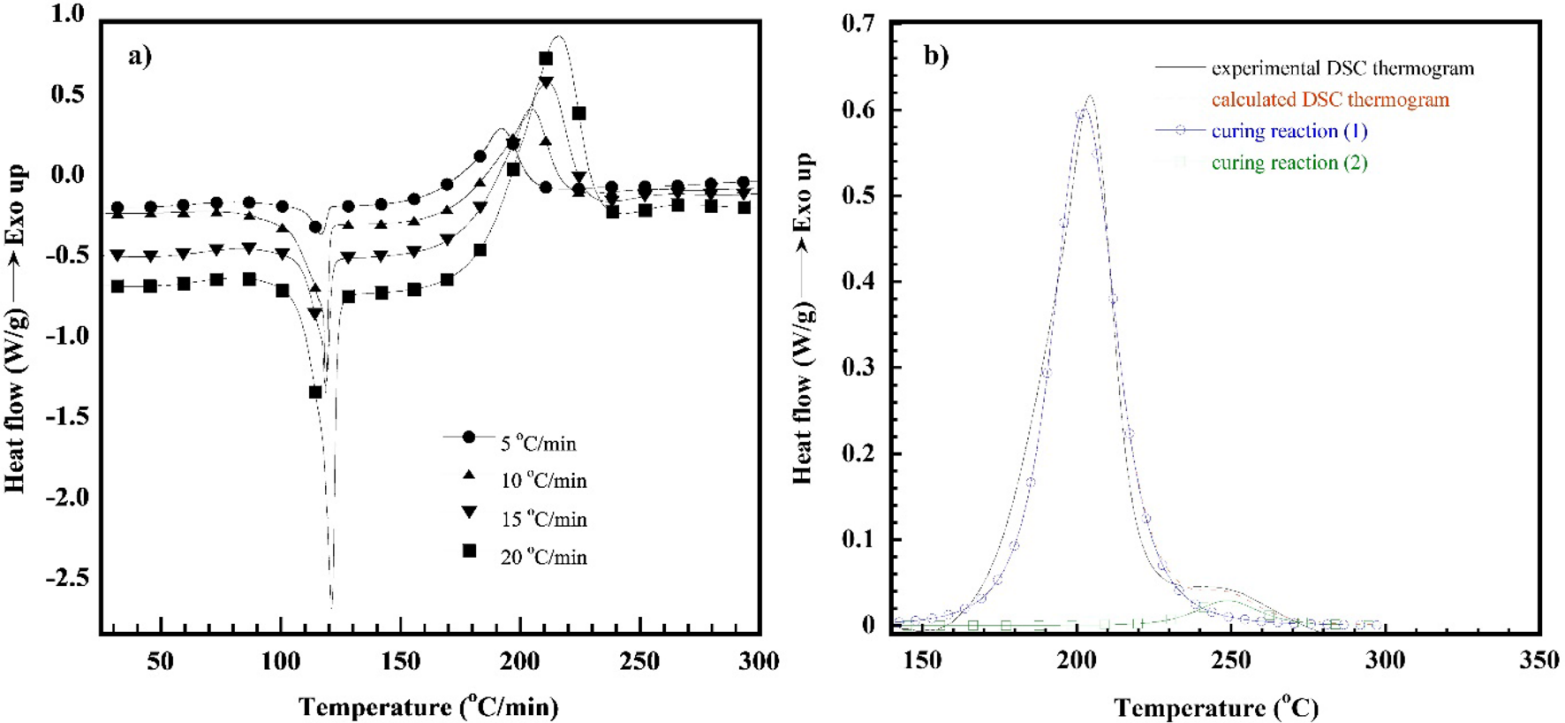
**a** DSC thermogram of V-fa/ECO (80/20) at various heating rates (β), **b** DSC thermogram of V-fa/ECO (80/20) recorded at 10 °C/min.

DSC thermograms of heat flow as a function of temperature of the V-fa/ECO (80/20) monomer mixture at a heating rate of 5, 10, 15, and 20 °C/min are plotted in Fig. 8a; these were used to evaluate the curing kinetic parameters, i.e., activation energy (*E*_*a*_), pre-exponential factor (*A*) and order of reaction (*n*, *m*). As above mentioned in Sect. "Curing behavior of V-fa/ECO monomer mixtures", the curing reaction of the V-fa/ECO monomer mixtures consisted of two exothermic curing peaks. Therefore, each exothermic curing peak of the V-fa/ECO (80/20) monomer mixture heated with different heating rates was separated by peaks fitting and deconvolution using Pearson VII distribution. For example, the separated exothermic curing peak of the V-fa/ECO (80/20) monomer mixture with a heating rate of 10 °C/min are presented in Fig. 8b.

Figure 8b shows the exothermic curing peaks of the V-fa/ECO (80/20) monomer mixture at peak-1 and peak-2 which were called curing reaction (1) and curing reaction (2), respectively; the curing kinetic parameters of each curing reaction were then evaluated. With two curing reaction pathways of the V-fa/ECO (80/20) monomer mixture, it is possible that its curing reaction tends to complicate that the activation energy may not unique. Therefore, the isoconversional methods, i.e., Flynn–Wall–Ozawa’s method (FWO) and Friedman’s method (FR) which completely evaluate the activation energy at different conversion levels regardless of the form of the kinetic equation was adopted in this work. The FWO is based on Eq. (1), while the FR is based on Eqs. (2) and (3) (Barros et al., 2020; Yousef et al., 2021):

1 $$ln\beta =ln\left(\frac{A{E}_{a}}{R}\right)-lng\left(\alpha \right)-5.331-1.052\left(\frac{{E}_{a}}{RT}\right)$$

When $$g\left(\alpha \right)={\int}_{0}^{\alpha }\frac{d\alpha }{f\left(\alpha \right)}$$

2 $$\frac{d\alpha }{dt}=A\left(\alpha \right)f\left(\alpha \right)exp\left(\frac{{E}_{a}}{RT}\right)$$

3 $$ln\frac{d\alpha }{dt}=ln\beta \frac{d\alpha }{dT}=ln\left[Af\left(\alpha \right)\right]-\frac{{E}_{a}}{RT}$$

where *β* is the heating rate, *E*_*a*_ is the activation energy at a given conversion (or apparent activation energy), *R* is the gas constant, *T* is the temperature at a certain conversion and *g*(*α*) is the integral conversion function.

**Fig. 9:**
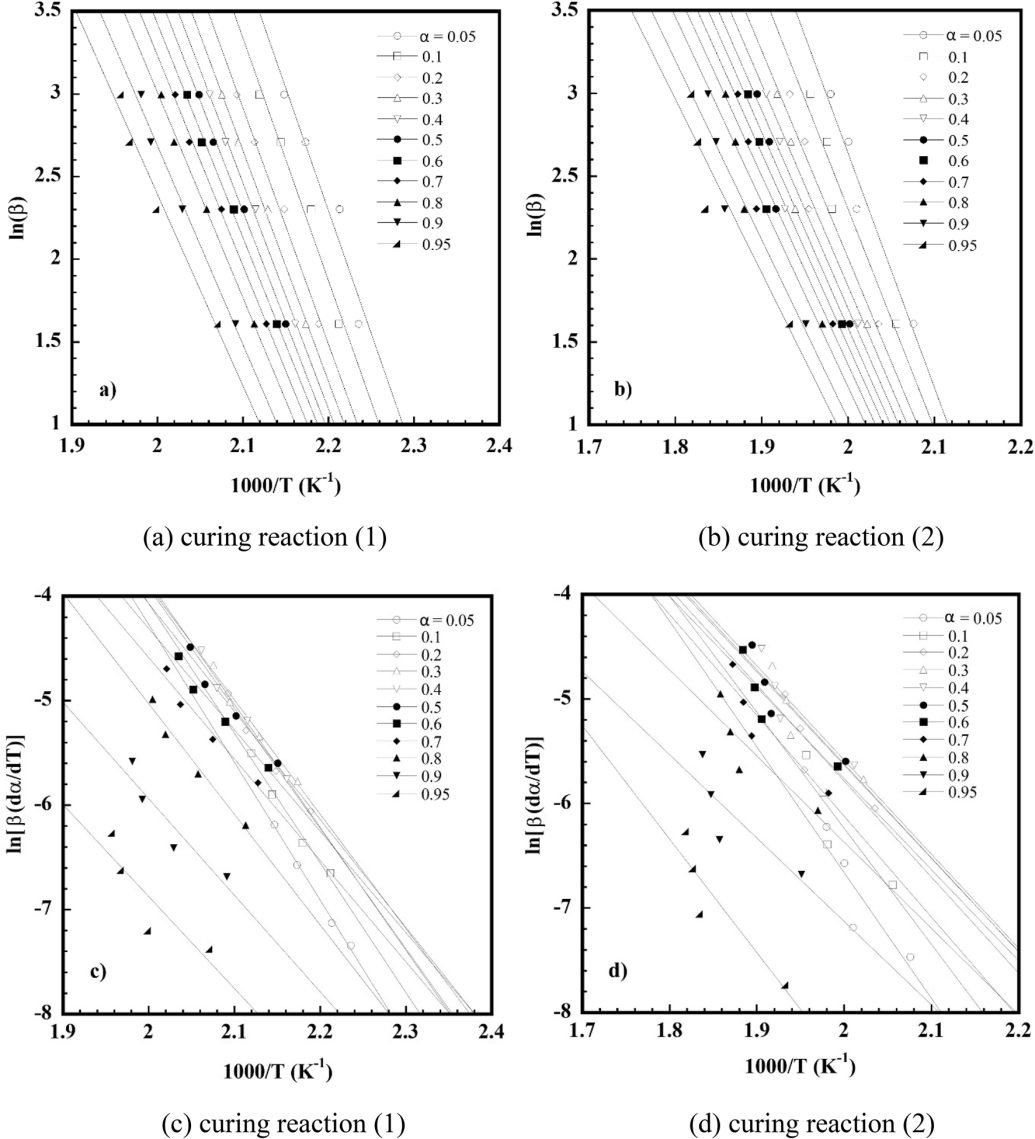
**a**, **b** FWO plots at various α of V-fa/ECO (80/20) monomer mixture for curing reaction (1) and curing reaction (2), **c**, **d** FR plots at various α of V-fa/ECO (80/20) monomer mixture for curing reaction (1) and curing reaction (2)

**Fig. 10:**
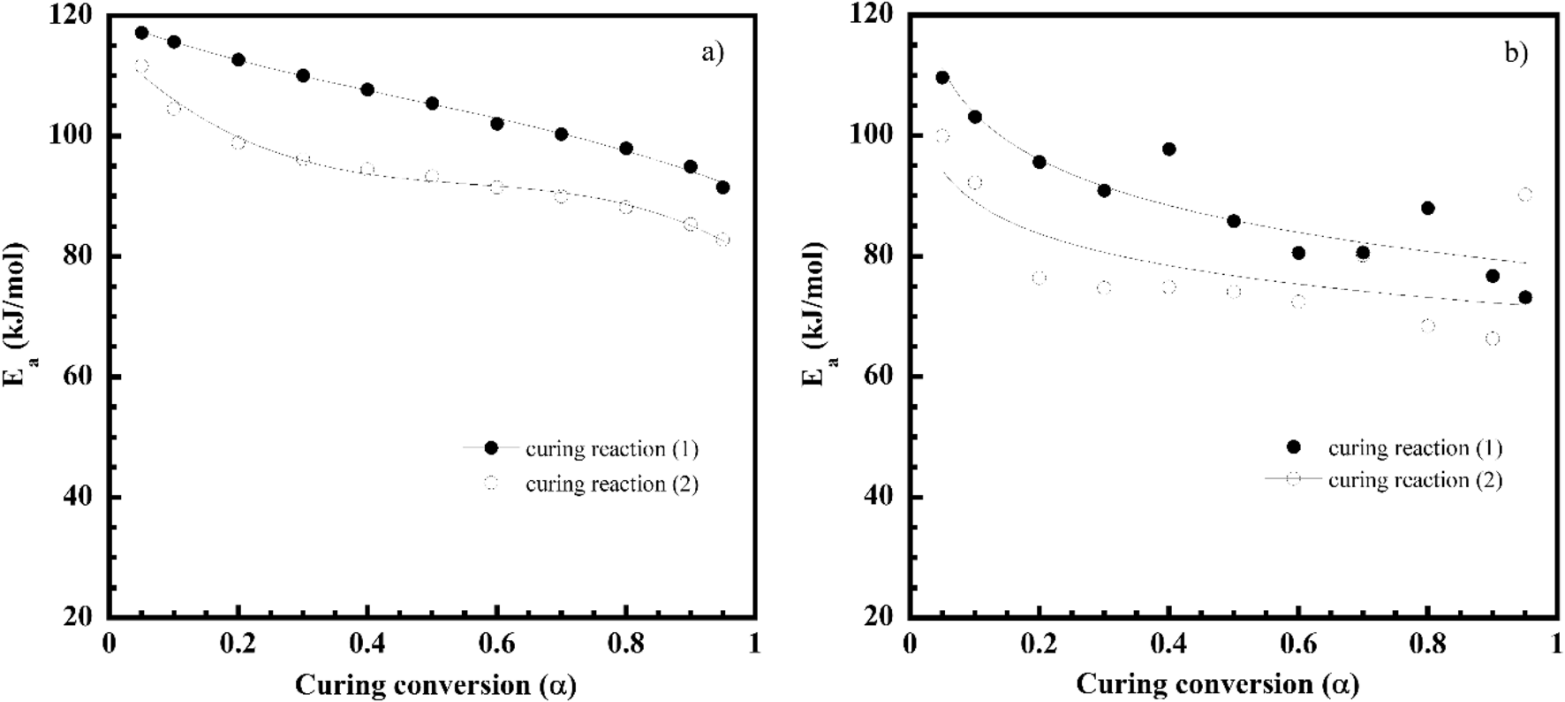
**a** *E*_*a*_ obtained from FWO, **b** *E*_*a*_ obtained from FR of V-fa/ECO (80/20) monomer mixture: (●) curing reaction (1) and (○) curing reaction (2)

Figure 9a, b plots the ln*β* values as a function of 1/*T* at each constant curing conversion (*α*) according to the FWO for each curing reaction of the V-fa/ECO (80/20) monomer mixture. The straight lines with the linear correlation coefficients, where the slope allows the evaluation of the *E*_*a*_ are plotted in Fig. 10a. The *E*_*a*_ of each curing reaction tended to decrease with an increase in the curing conversion throughout the entire curing reaction. For the curing reaction (1), the autocatalytic effect was characteristic, resulting from the hydroxyl groups generated from ring opening of the V-fa monomer by heat to promote the oxazine ring opening of the V-fa, leading to a decrease in the *E*_*a*_ as similarly observed for the guaiacol bio-based benzoxazine system (Ručigaj et al. 2016). In addition, it is also possible that viscosity of the V-fa/ECO (80/20) monomer mixture was reduced due to an increase of temperature during the process of the curing reaction, the elevated mobility of the chain segments of the monomer mixture and the growing effective collision of molecular reaction groups further also accelerated the diffusion rate and reduce the activation energy (Roudsari et al. 2014; Wu et al. 2018). For the curing reaction (2), this phenomenon is due to the fact that the generated hydroxyl group of the V-fa monomer partially protonates the oxygen atom of the epoxy group, causing a ring-opening reaction, resulting in a hydroxyl group (Jubsilp et al. 2006, 2010; Shutov et al. 2022). The hydroxyl groups formed by oxirane ring opening have a self-accelerating effect on the curing reaction (2) and lower activation energy values (Wu et al. 2018).

Figure 9c, d shows the relationship between ln[*β*(*dα/dT*)] and 1/*T* at constant *α* for a set of heating rates (*β*) according to the FR method. A family of straight line with slope of -*E*_*a*_/*R* was obtained. The *E*_*a*_ as a function of *α* is plotted in Fig. 10b. It can be seen a decrease of the *E*_*a*_ with conversion like that observed in the FWO. As it was mentioned the FWO method is potentially suited for use in curing systems, where many reactions are occurring having not very different activation energies which varies with time. Therefore, in this work, the average value of the activation energy of curing reaction (1) and curing reaction (2) of the V-fa/ECO (80/20) monomer mixture experimentally determined by the FWO were used to determine the model. The average activation energy based on the FWO of the curing reaction (1) and the curing reaction (2) was 105 kJ/mol and 94 kJ/mol, respectively.

To confirm that the curing reaction (1) and curing reaction (2) are autocatalytic curing reaction, the curing reaction model of the V-fa/ECO (80/20) monomer mixture was investigated by the FR. In case of the *n*th-order reaction, *f*(*α*) = (*1*−*α*)^n^ is substituted in Eqs. (3) and (4) is obtained. The slope corresponds to the reaction order (*n*):

4 $$ln\left[Af\left(\alpha \right)\right]=ln\left(\frac{d\alpha }{dt}\right)+\frac{{E}_{a}}{RT}=lnA+nln\left(1-\alpha \right)$$

**Fig. 11:**
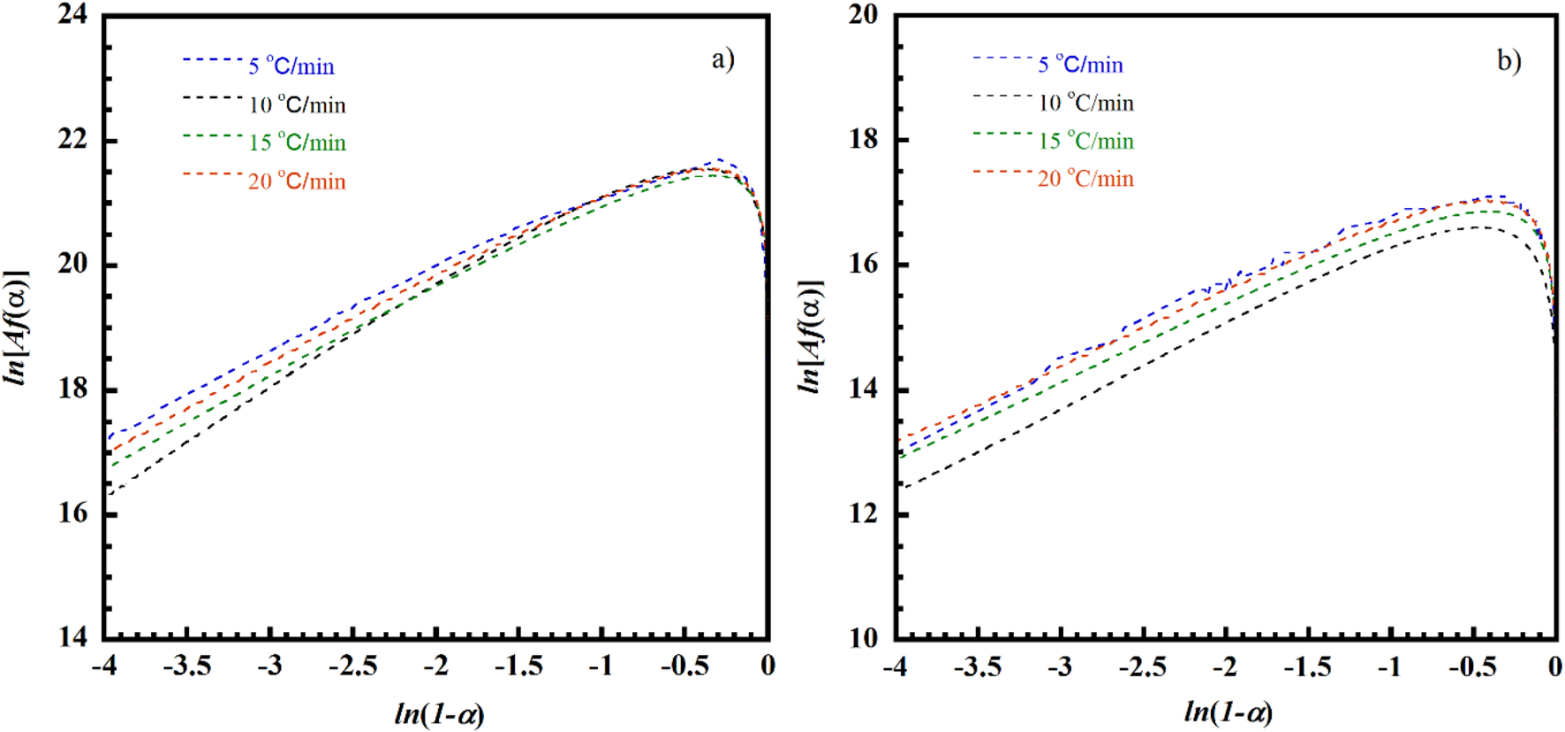
Plots of *ln*[*Af*(*α*)] vs. *ln*(*1 − α*) of V-fa/ECO (80/20) monomer mixture at various heating rates and using average *E*_*a*_ from the FWO: **a** curing reaction (1) and **b** curing reaction (2)

Figure 11 shows the plot of *ln*[*Af*(*α*)], which are summation of *ln*(*dα*/*dt*) and *E*_*a*_/*RT*, and *ln*(*1−α*) of the curing reactions (1) and the curing reaction (2) of the V-fa/ECO (80/20) monomer mixture at a heating rate of 5, 10, 15, and 20 °C/min. The *E*_*a*_/*RT* value can be obtained using the average *E*_*a*_ from the FWO. The *ln*[*Af*(*α*)] and *ln*(*1−α*) are not linearly related and evidently show a maximum in the range of *ln*(*1−α*) approximately around 0.51 to 0.22 which is equivalent to curing conversion (*α*) of about 0.2–0.4 (Barros et al. 2020); this suggests that both curing reactions are autocatalytic curing process.

The autocatalytic curing reaction model considers reaction orders: *m* and *n*, as shown in Eq. (5) and by taking the logarithm of Eq. (6), a linear expression for the logarithm of curing rate can be obtained:

5 $$\frac{d\alpha }{dt}=Aexp\left(-\frac{{E}_{a}}{RT}\right){\left(1-\alpha \right)}^{n}{\alpha }^{m}$$

6 $$ln\left(\beta \frac{d\alpha }{dt}\right)=lnA-\left(\frac{{E}_{a}}{RT}\right)+nln\left(1-\alpha \right)+mln\left(\alpha \right)$$

Equation (6) can be solved by multiple linear regression. The *A*, *m*, and *n* values of two curing reactions can be obtained using the average *E*_*a*_ from the FWO. The degree of curing is in a range of 0.1–0.95. The results of the multiple linear regressions analysis for all heating rates of the V-fa/ECO (80/20) monomer mixture for the curing reaction (1) and the curing reaction (2) are listed in Tables 1 and 2, respectively.

**Table 1:** Kinetic parameters evaluated for the curing of the V-fa/ECO (80/20) (curing reaction (1))

Heating rate (**°**C/min)	*E*_*a*_ (kJ/mol)	ln*A* (s^−1^)	Mean	*n*	Mean	*m*	Mean
5	105	22.68	22.80	1.54	1.50	1.08	0.80
10		23.04		1.57		0.79	
15		22.65		1.45		0.58	
20		22.81		1.44		0.72

**Table 2:** Kinetic parameters evaluated for the curing of the V-fa/ECO (80/20) (curing reaction (2))

Heating rate (°C/min)	*E*_*a*_ (kJ/mol)	ln*A* (s^−1^)	Mean	*n*	Mean	*m*	Mean
5	94	18.95	18.40	1.68	1.44	1.26	0.88
10		18.15		1.46		0.88	
15		18.14		1.34		0.68	
20		18.35		1.29		0.72	

Consequently, we obtain the mathematical models for autocatalytic kinetics of the V-fa/ECO (80/20) for curing reaction (1) and curing reaction (2) as

7 $$\frac{d\alpha }{dt}=1.13\times {10}^{10}Aexp\left(-\frac{12629}{T}\right){\left(1-\alpha \right)}^{1.5}{\alpha }^{0.8}$$

8 $$\frac{d\alpha }{dt}=1.15\times {10}^{8}Aexp\left(-\frac{11306}{T}\right){\left(1-\alpha \right)}^{1.44}{\alpha }^{0.88}$$

**Fig. 12:**
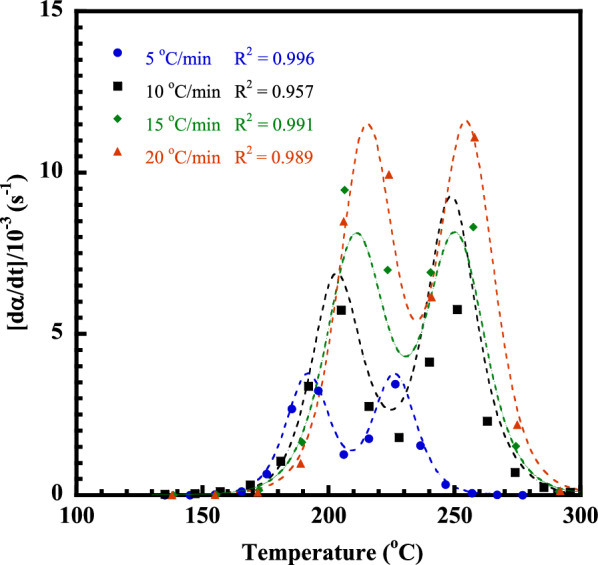
Comparison of the experimental (symbols) and calculated (solid lines) DSC peaks corresponding to the total curing process of V-fa/ECO (80/20) monomer mixture at different heating rates

The experimental DSC peaks were compared with the calculated data from the models for the V-fa/ECO (80/20) monomer mixture, as shown in Fig. 12. It is clear that the data calculated from the model are in good agreement with the experimental data. In addition, it was observed that bio-based benzoxazine/epoxy monomer system still showed two curing reactions similar to that of the petroleum-based one (Shutov et al. 2022). The obtained activation energy of the V-fa/ECO (80/20) monomer mixture which is the bio-based system was in the same range of the petroleum-based benzoxazine/epoxy system such as bisphenol A/aniline-based benzoxazine (BA-a) blended with bisphenol A/epichlorohydrin (epoxy).

### Feasibility of glass fiber (GF)-reinforced V-f/ECO biocopolymer for dental fiber post

To compare the developed 65.4 vol% GF-reinforced V-fa/ECO (80/20) biocopolymer post against a commercial GF post, the stress distribution was investigated using finite-element analysis (FEA) on a restored tooth model by applying the 100 N oblique load at 45 degree. The elastic properties which are the key material properties for numerical simulation of the isotropic materials and the glass fiber posts (orthotropic materials) used for the FEA are listed in Tables 3 and 4, respectively.

**Table 3:** Elastic properties of the isotropic materials used for the FEA

Materials	Elastic modulus (GPa)	Poisson’s coefficient
Porcelain (crown) (Genovese et al. 2005; Ko et al. 1992; Pegoretti et al. 2002)	120	0.28
Composite resin (Chatvanitkul & Lertchirakarn 2010; Willems et al. 1992)	16.6	0.24
Gutta-percha (Genovese et al. 2005; Ko et al. 1992; Pegoretti et al. 2002)	0.00069	0.45
Dentin (Ko et al. 1992; Pegoretti et al. 2002)	18.6	0.31
Cortical bone (Ko et al. 1992; Pegoretti et al. 2002)	13.7	0.30
Periodontal ligament (Pegoretti et al. 2002)	0.0689	0.45
Cancellous bone (Pegoretti et al. 2002)	1.37	0.30

**Table 4:** Elastic properties for the orthotropic materials used for the FEA

Elastic constant	GF-reinforced V-fa/ECO biocopolymer post (65.4 vol%)	Commercial GF post (Coelho et al. 2009)
*E*_L_ (GPa)	21.47	37.00
*E*_T_ = *E*_T′_ (GPa)	10.54	9.50
*G*_LT_ = *G*_LT′_ (GPa)	7.50	3.10
*G*_TT′_ (GPa)	4.22	3.50
ν_LT_ = ν_LT′_	0.22	0.27
ν_TL_ = ν_T′L_	0.11	0.34
ν_TT′_	0.25	0.27

*E*_L_ obtained from experimental data from flexural test, while the other values are theoretical values calculated from relationships as shown in Appendix A in Pegoretti et al. 2002: E (Elastic modulus), G (Shear modulus), ν (Poisson ratios), L (Longitudinal direction), T and T′ (Transverse direction)

**Fig. 13:**
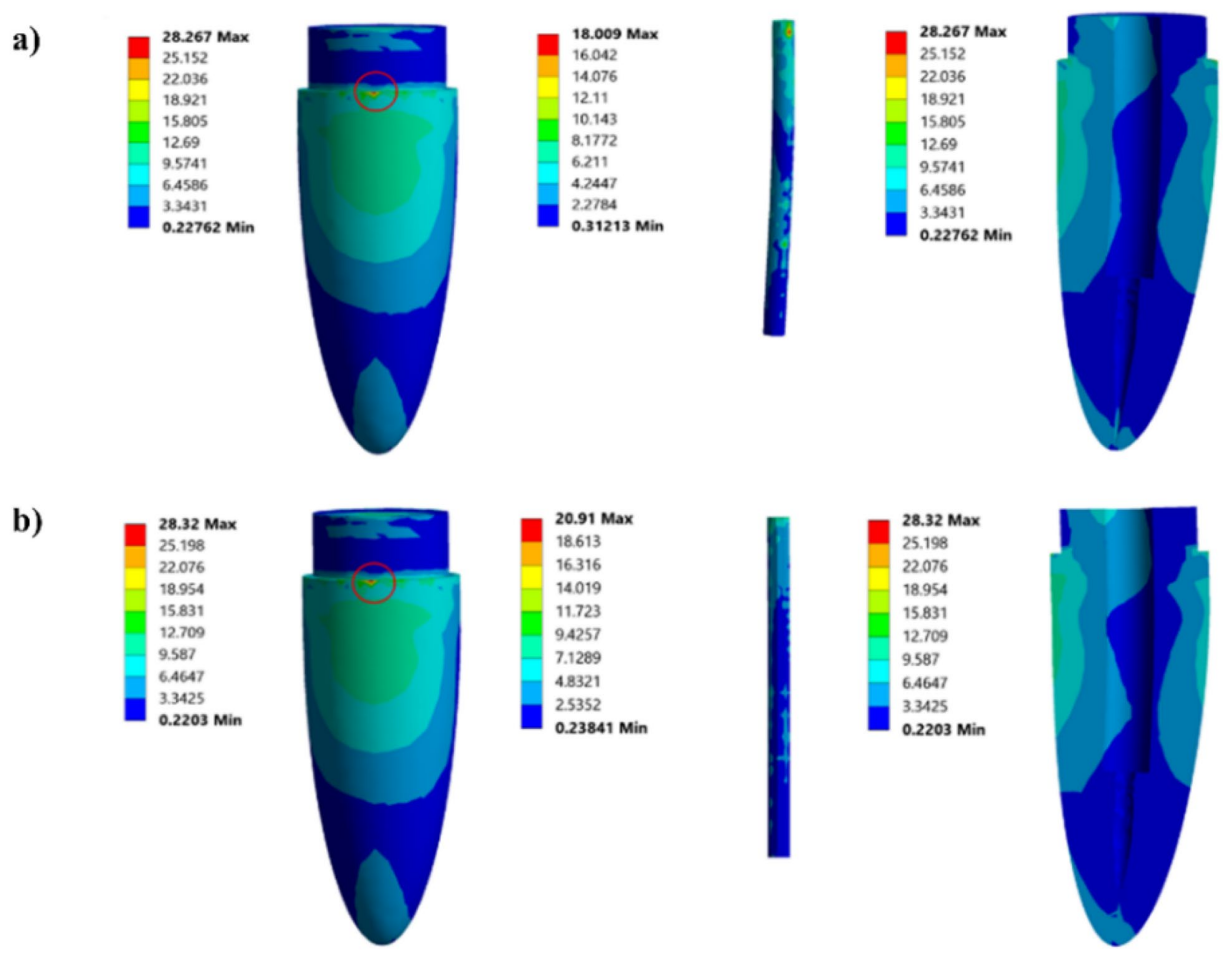
Contour map of the von Mises stress of tooth restored with glass fiber posts: **a** 65.4 vol% glass fiber-reinforced poly(V-fa)/ECO biocopolymer post, **b** commercial glass fiber post

The von Mises stress (σ_v_) contour maps of a tooth model restored with the GF-reinforced V-fa/ECO biocopolymer post are presented in Fig. 13a and of a commercially available glass fiber post in Fig. 13b. It was noticed that both tooth models exhibited the maximum σ_v_ in the cervical region in dentin. In addition, the tooth model restored with the GF-reinforced V-fa/ECO biocopolymer post showed lower value of the maximum σ_v_ at the dental region, i.e., 28.267 MPa than that of the tooth model restored with commercial glass fiber post, i.e., 28.320 MPa. It is possible that the GF-reinforced V-fa/ECO biocopolymer post allowed to carry load fractions, since its elastic modulus of 21.47 GPa as reported in Table 4 showed closer to dentin with the elastic modulus approximately 18–20 GPa than the elastic modulus of the commercial glass fiber post, i.e., 37.00 GPa (Ko et al. 1992; Pegoretti et al. 2002; Teshigawara et al. 2019). Therefore, this characteristic indicated that the chance of fracture of the root canal can be reduced by the utilization of the GF-reinforced V-fa/ECO biocopolymer as a glass fiber post.

## Conclusions

Benzoxazine–epoxy biocopolymer was successfully proved to act as a matrix for dental fiber post application. The obtained benzoxazine–epoxy biocopolymer showed good mechanical and thermal properties, i.e., storage modulus of 2.27 GPa, a T_g_ at 158 °C and T_d5_ of 323 °C. The curing reaction of V-fa/ECO monomer mixture indicated several reactions presenting overlapped peaks: the catalytic opening of oxazine ring and an epoxy group, the autocatalytic reaction of benzoxazine and etherification between benzoxazine and epoxide, and epoxy homopolymerization which are quite similar to reactions of petroleum-based benzoxazine–epoxy copolymer. Its curing kinetics were studied using the Flynn–Wall–Ozawa method and were derived assuming constant *E*_a_. The kinetic equation of curing was obtained at $\frac{d\alpha }{dt}=1.13\times {10}^{10}exp\left(-\frac{105000}{RT}\right){\left(1-\alpha \right)}^{1.5}{\alpha }^{0.8}$ for curing reaction (1) and at $\frac{d\alpha }{dt}=1.15\times {10}^{8}exp\left(-\frac{94000}{RT}\right){\left(1-\alpha \right)}^{1.44}{\alpha }^{0.88}$ for curing reaction (2). For glass fiber-reinforced V-fa/ECO biocopolymer acted as a dental fiber post, the proper proportion blending V-fa with ECO could be employed in dentistry as the matrix of fiber-reinforced dental root canal posts; the glass fiber-reinforced V-fa/ECO biocopolymer post has an elastic modulus that more closely approaches that of dentin while that for commercial glass fiber post was much higher. Moreover, based on this work, the glass fiber-reinforced V-fa/ECO biocopolymer may provide in future applications that requires good mechanical property and eco-friendly materials.

## Data Availability

All data analyzed during this study are included in this article.

## References

[CR1] Ambrozic R, Rucigaj A, Krajnc M (2020). A green approach toward epoxy-benzoxazine copolymers with shape-memory ability. Express Polym Lett.

[CR2] Amornkitbamrung L, Srisaard S, Jubsilp C, Bielawski CW, Um SH, Rimdusit S (2020). Near-infrared light responsive shape memory polymers from bio-based benzoxazine/epoxy copolymers produced without using photothermal filler. Polymer.

[CR3] Barros JJP, Silva, I.D.d.S., Jaques, N.G., Wellen, R.M.R.  (2020). Approaches on the non-isothermal curing kinetics of epoxy/PCL blends. J Mater Res Technol.

[CR4] Bhardwaj N, Kumar B, Agrawal K, Verma P (2021). Current perspective on production and applications of microbial cellulases: a review. Bioresour Bioprocess.

[CR5] Birniwa AH, Abdullahi SS, Ali M, Mohammad RE, Jagaba AH, Amran M, Avudaiappan S, Maureira-Carsalade N, Flore EIS (2023). Recent trends in treatment and fabrication of plant-based fiber-reinforced epoxy composite: a review. J Compos Sci.

[CR6] Cao J, Duan H, Zou J, Zhang J, Wan C, Zhang C, Ma H (2022). Bio-based phosphorus-containing benzoxazine towards high fire safety, heat resistance and mechanical properties of anhydride-cured epoxy resin. Polym Degrad Stabil.

[CR7] Chatvanitkul C, Lertchirakarn V (2010). Stress distribution with different restorations in teeth with curved roots: a finite element analysis study. J Endod.

[CR8] Chen X, Yuan L, Zhang Z, Wang H, Liang G, Gu A (2015). New glass fiber/bismaleimide composites with significantly improved flame retardancy, higher mechanical strength and lower dielectric loss. Compos B Eng.

[CR9] Coelho C, Biffi J, Silva G, Abrahao A, Campos R, Soares C (2009). Finite element analysis of weakened roots restored with composite resin and posts. Dent Mater J.

[CR10] Costa TS, Brandão RMR, Farias Vajgel BC, SoutoMaior JR (2022) CAD-CAM glass fiber compared with conventional prefabricated glass fiber posts: A systematic review. *Journal Prosthet Dent*10.1016/j.prosdent.2022.01.007 35933174

[CR11] De Luca MA, Martinelli M, Barbieri CCT (2009). Hybrid films synthesised from epoxidised castor oil, γ-glycidoxypropyltrimethoxysilane and tetraethoxysilane. Prog Org Coat.

[CR12] Dutta T, Ghosh NN, Das M, Adhikary R, Mandal V, Chattopadhyay AP (2020). Green synthesis of antibacterial and antifungal silver nanoparticles using Citrus limetta peel extract: experimental and theoretical studies. J Environ Chem Eng.

[CR13] Dutta T, Chowdhury SK, Ghosh NN, Chattopadhyay AP, Das M, Mandal V (2022). Green synthesis of antimicrobial silver nanoparticles using fruit extract of Glycosmis pentaphylla and its theoretical explanations. J Mol Struct.

[CR14] Elsubeihi E, Aljafarawi T, Elsubeihi H (2020). State of the art contemporary prefabricated fiber-reinforced posts. Open Dent J.

[CR15] Forchetti Casarino A, Bortolato SA, Casis N, Estenoz DA, Spontón ME (2023). Novel polybenzoxazine and polybenzoxazine/epoxy thermosetting copolymers containing polysilsesquioxane nanostructures for high-performance thermal protection systems. Eur Polym J.

[CR16] Fouad H, Mourad A-HI, Alshammari BA, Hassan MK, Abdallah MY, Hashem M (2020). Fracture toughness, vibration modal analysis and viscoelastic behavior of Kevlar, glass, and carbon fiber/epoxy composites for dental-post applications. J Mech Behav Biomed Mater.

[CR17] Genovese K, Lamberti L, Pappalettere C (2005). Finite element analysis of a new customized composite post system for endodontically treated teeth. J Biomech.

[CR18] Gotro, J. (2022) Epoxy Curing agents – anhydrides, long pot life and exceptional properties. https://polymerinnovationblog.com. Accessed 3 Jul 2023.

[CR19] Hara, O. (1990) Curing agents for epoxy resin. https://www.threebond.co.jp/en/technical/technicalnews/pdf/tech32.pdf. Accessed 3 Jul 2023

[CR20] Hombunma P, Parnklang T, Mora P, Jubsilp C, Rimdusit S (2019). Shape memory polymers from bio-based benzoxazine/epoxidized natural oil copolymers. Smart Mater Struct.

[CR21] Jubsilp C, Damrongsakkul S, Takeichi T, Rimdusit S (2006). Curing kinetics of arylamine-based polyfunctional benzoxazine resins by dynamic differential scanning calorimetry. Thermochim Acta.

[CR22] Jubsilp C, Punson K, Takeichi T, Rimdusit S (2010). Curing kinetics of Benzoxazine–epoxy copolymer investigated by non-isothermal differential scanning calorimetry. Polym Degra Stab.

[CR23] Jubsilp C, Panyawanitchakun C, Rimdusit S (2013). Flammability and thermomechanical properties of dianhydride-modified polybenzoxazine composites reinforced with carbon fiber. Polym Compos.

[CR24] Ko CC, Chu CS, Chung KH, Lee MC (1992). Effects of posts on dentin stress distribution in pulpless teeth. J Prosthet Dent.

[CR25] Kurihara S, Idei H, Aoyagi Y, Kuroe M (2012) Binder resin for friction material, binder resin composition for friction material, composite material for friction material containing the same, friction material and production method thereof. US8227390

[CR26] Leong YK, Show PL, Lin HC, Chang CK, Loh H-S, Lan JC-W, Ling TC (2016). Preliminary integrated economic and environmental analysis of polyhydroxyalkanoates (PHAs) biosynthesis. Bioresour Bioprocess.

[CR27] Li S, Yan H, Feng S, Niu S (2015). Synthesis and characterization of a phosphorus-containing flame retardant with double bonds and its application in bismaleimide resins. RSC Adv.

[CR28] Liu F, Wang Z, Liu D, Li J (2009). Curing of diglycidyl ether of bisphenol-A epoxy resin using a poly(aryl ether ketone) bearing pendant carboxyl groups as macromolecular curing agent. Polym Inter.

[CR29] Mora P, Nunwong C, Sriromreun P, Kaewsriprom P, Srisorrachatr U, Rimdusit S, Jubsilp C (2022). High performance composites based on highly filled glass fiber-reinforced polybenzoxazine for post application. Polymers.

[CR30] Nie S, Jin D, Xu Y, Han C, Dong X, Yang J-N (2020). Effect of a flower-like nickel phyllosilicate-containing iron on the thermal stability and flame retardancy of epoxy resin. J Mater Res Technol.

[CR31] Okhawilai M, Dueramae I, Jubsilp C, Rimdusit S (2017). Effects of high nano-SiO2 contents on properties of epoxy-modified polybenzoxazine. Polym Compos.

[CR32] Ortiz-Magdaleno M, Bogarin-Topete ER, Cerda-Cristerna BI, Gutiérrez-Sánchez M (2023) Effect of degree of conversion on the surface properties of polymerized resin cements used for luting glass fiber posts. *J Prosthet Dent*10.1016/j.prosdent.2023.05.009 37357085

[CR33] Park S-J, Jin F-L, Lee J-R (2004). Synthesis and thermal properties of epoxidized vegetable oil. Macromol Rapid Commun.

[CR34] Pegoretti A, Fambri L, Zappini G, Bianchetti M (2002). Finite element analysis of a glass fibre reinforced composite endodontic post. Biomaterials.

[CR35] Prasomsin W, Parnklang T, Sapcharoenkun C, Tiptipakorn S, Rimdusit S (2019). Multiwalled carbon nanotube reinforced bio-based benzoxazine/epoxy composites with NIR-laser stimulated shape memory effects. Nanomaterials.

[CR36] Rao BS, Rajavardhana Reddy K, Pathak SK, Pasala AR (2005). Benzoxazine–epoxy copolymers: effect of molecular weight and crosslinking on thermal and viscoelastic properties. Polym Inter.

[CR37] Rimdusit S, Ishida H (2000). Synergism and multiple mechanical relaxations observed in ternary systems based on benzoxazine, epoxy, and phenolic resins. Polym Sci Polym Phys.

[CR38] Roudsari G, Mohanty A, Misra M (2014). Study of the curing kinetics of epoxy resins with biobased hardener and epoxidized soybean oil. ACS Sustain Chem Eng.

[CR39] Ručigaj A, Gradišar Š, Krajnc M (2016). Kinetic investigation of a complex curing of the guaiacol bio-based benzoxazine system. E-Polymers.

[CR40] Saba N, Jawaid M, Alothman OY, Almutairi Z (2019). Evaluation of dynamic properties of nano oil palm empty fruit bunch filler/epoxy composites. J Mater Res Technol.

[CR41] Shadhin M, Rahman M, Jayaraman R, Mann D (2021). Novel cattail fiber composites: converting waste biomass into reinforcement for composites. Bioresour Bioprocess.

[CR42] Shutov VV, Bornosuz NV, Korotkov RF, Gorbunova IY, Sirotin IS (2022). Kinetics of benzoxazine and epoxy oligomer copolymerization. Thermochim Acta.

[CR43] Shuvo II (2020). Fibre attributes and mapping the cultivar influence of different industrial cellulosic crops (cotton, hemp, flax, and canola) on textile properties. Bioresour Bioprocess.

[CR44] Sini NK, Bijwe J, Varma IK (2014). Renewable benzoxazine monomer from vanillin: synthesis, characterization, and studies on curing behavior. J Polym Sci Polym Chem.

[CR45] Srisaard S, Amornkitbamrung L, Charoensuk K, Sapcharoenkun C, Jubsilp C, Rimdusit S (2021). Effects of graphene nanoplatelets on bio-based shape memory polymers from benzoxazine/epoxy copolymers actuated by near-infrared light. J Intell Mater Syst Struct.

[CR46] Sudha GS, Kalita H, Mohanty S, Nayak SK (2017). Biobased epoxy blends from epoxidized castor oil: effect on mechanical, thermal, and morphological properties. Macromol Res.

[CR47] Teshigawara D, Ino T, Otsuka H, Isogai T, Fujisawa M (2019). Influence of elastic modulus mismatch between dentin and post-and-core on sequential bonding failure. J Prosthodont Res.

[CR48] Trapé DV, López OV, Villar MA (2021). Vinasse: from a residue to a high added value biopolymer. Bioresour Bioprocess.

[CR49] Wang Y, Wang S, Meng Y, Liu Z, Li D, Bai Y, Yuan G, Wang Y, Zhang X, Li X, Deng X (2022) Pyro-catalysis for tooth whitening via oral temperature fluctuation. Nat Commun 13:4419 10.1038/s41467-022-32132-3 PMC9338087 35906221

[CR50] Willems G, Lambrechts P, Braem M, Celis JP, Vanherle G (1992). A classification of dental composites according to their morphological and mechanical characteristics. Dent Mater.

[CR51] Wu F, Zhou X, Yu X (2018). Reaction mechanism, cure behavior and properties of a multifunctional epoxy resin, TGDDM, with latent curing agent dicyandiamide. RSC Adv.

[CR52] Yousef S, Eimontas J, Striūgas N, Subadra SP, Abdelnaby MA (2021). Thermal degradation and pyrolysis kinetic behaviour of glass fibre-reinforced thermoplastic resin by TG-FTIR, Py-GC/MS, linear and nonlinear isoconversional models. J Mater Res Techonol.

[CR53] Zeng C, Lu S, Song L, Xiao X, Gao J, Pan L, He Z, Yu J (2015). Enhanced thermal properties in a hybrid graphene-alumina filler for epoxy composites. RSC Adv.

[CR54] Zhang Y, Duan C, Bokka SK, He Z, Ni Y (2022). Molded fiber and pulp products as green and sustainable alternatives to plastics: a mini review. J Bioresour Bioprod.

[CR55] Zulkifli NN, Badri KH, Amin KAM (2016). Palm kernel oil-based polyester polyurethane composites incorporated with multi-walled carbon nanotubes for biomedical application. Bioresour Bioprocess.

